# 基于Oncomine数据库荟萃分析KIF23在非小细胞肺癌中的表达和意义

**DOI:** 10.3779/j.issn.1009-3419.2017.12.05

**Published:** 2017-12-20

**Authors:** 亮 叶, 慧娟 李, 方 张, 镗烽 吕, 红兵 刘, 勇 宋

**Affiliations:** 1 210006 南京，南京医科大学金陵临床医学院呼吸内科 Department of Respiratory Medicine, Jinling Clinical Medical College of Nanjing Medical University, Nanjing 210006, China; 2 210002 南京，南京医科大学附属南京医院（南京市第一医院）呼吸内科 Department of Respiratory Medicine, Najing Hospital Affiliated to Nanjing Medical University (Nanjing First Hospital), Nanjing 210002, China

**Keywords:** 肺肿瘤, 驱动蛋白超家族23, Oncomine, Lung neoplasms, Kinesin family member 23, Oncomine

## Abstract

**背景与目的:**

非小细胞肺癌（non-small cell lung cancer, NSCLC）是全球引起死亡的最重要原因之一。大多数患者发现时处于中晚期，预后较差。本研究拟探讨驱动蛋白超家族23（kinesin family member 23, KIF23）在NSCLC中的表达及意义。

**方法:**

收集Oncomine数据库中关于KIF23的信息，并对目前数据库中资料进行二次分析，对其在NSCLC中的作用进行荟萃分析。利用*Kaplan*-*Meier* Plotter进行患者生存周期分析。

**结果:**

Oncomine数据库中共收集了447项不同类型的研究结果，其中关于KIF23表达有统计学差异的研究结果有67个，KIF23表达增高的研究有64项、表达降低的研究有3项。共有16项研究涉及KIF23在NSCLC癌组织和正常组织中的表达，共包括1, 189个样本，与对照组相比，KIF23在NSCLC细胞癌中高表达（*P*＜0.05）。不仅如此，KIF23表达量与NSCLC总体生存率存在相关性，高表达KIF23的患者总体生存率较差，低表达KIF23的患者预后较好（*P*＜0.05）。进一步亚组分析发现，KIF23表达水平对肺腺癌患者预后有显著影响，而在鳞癌患者中，其表达水平对预后无显著影响。

**结论:**

我们通过对Oncomine基因芯片数据库中肿瘤相关基因信息的深入挖掘，提出KIF23在NSLCL组织中高表达，且与NSCLC预后有关，可能为肿瘤药物的开发提供重要理论依据。

## Abstract (en)

**Background and objective:**

Non-small cell lung cancer (NSCLC) is one of the most common cause of cancer-related death worldwide. As the overall prognosis for affected patients is still poor, there is a need for biomarkers for prediction of survival and guiding individual therapy. This study is to explore the expression and significance of kinesin family member 23 (KIF23) in NSCLC.

**Methods:**

KIF23 data were retrieved from Oncomine NSCLC database. The prognostic value of KIF23 was retrieved from an online survival analysis tool "*Kaplan*-*Meier* Plotter" (KM plotter) database.

**Results:**

In Oncomine database, there were 447 studies of different types concerning expression of KIF23, of which 67 studies were of statistically significance (64 up-regulated and 3 down-regulated). A total of 16 studies were involved KIF23 in NSCLC tissues and normal tissues, including a total of 1, 189 samples. Overall, KIF23 expression in NSCLC is higher than that in normal tissues (*P* < 0.05). Moreover, *Kaplan*-*Meier* plots of overall survival indicated that KIF23 high expression is closely associated with poor survival in NSCLC (*P* < 0.05). Subgroup analysis revealed that KIF23 expression showed negative relation to the prognosis of pulmonary adenocarcinoma patients. Whereas, in those with squamous carcinoma KIF23 expression showed no effects on the prognosis of the patients.

**Conclusion:**

KIF23 was found highly expressed in NSCLC, which might be a marker for NSCLC prognosis.

非小细胞肺癌（non-small cell lung cancer, NSCLC）严重威胁我国人民健康，是致死率最高的恶性肿瘤之一，给社会造成了巨大的经济负担^[1, 2]^。虽然近年发现了许多新的治疗方式，但发现时大多处于中晚期，预后仍未得到显著改善^[3]^。从分子水平研究NSCLC发生发展的机制有利于发现新的分子靶点，开发新的治疗手段，对于减少患者痛苦和延长患者生存时间极为重要^[4]^。

Oncomine数据库是目前全球最大的癌基因芯片数据库和整合数据挖掘平台，旨在挖掘癌症基因信息。通过大学或研究机构邮箱可以免费注册使用。迄今为止，该数据库已经收集了715个基因表达数据集，86, 733个癌症组织和正常组织的样本数据。利用Oncomine数据库可以比较常见癌症类型和各自正常组织的差异表达分选，也可以探索各种癌症亚型以及基于临床和病理学的分析，进行差异表达分选、共表达分析，查找某中癌症中差异表达的基因，确定目的基因，进而确定研究方向，不仅可以节省科研成本，而且其信息也更加全面。

驱动蛋白超家族（kinesin family member, KIF）是一类基于微管的分子马达蛋白，介导多种功能，其异常表达，在肿瘤的发生发展过程中扮演重要角色^[5, 6]^。*KIF23*基因隶属于KIF家族，研究发现，其在有丝分裂的胞质分离过程中起着重要作用，其表达缺失可导致有丝分裂停滞^[7]^。尽管既往研究表明KIF23在多种肿瘤组织和细胞中表达增高^[8, 9]^，但是在非小细胞肺癌组织中鲜有系统研究。

本研究利用Oncomine数据库和*Kaplan*-*Meier* Plotters数据库分析KIF23在NSCLC中的表达和预后，通过二次分析荟萃KIF23和NSCLC可能存在的关系，为进一步研究KIF23在NSCLC发生发展的作用机制提供线索和依据，同时也为利用Oncomine数据库挖掘肿瘤基因学数据提供良好范例。

## 1 资料与方法

### 1.1 Oncomine数据库提取数据

Oncomine数据库是一个基于基因芯片的数据库和整合数据挖掘平台，在此数据中可根据自己的需求设定筛选和挖掘数据的条件。本研究中，我们设定的筛选条件为：①“Cancer Type: Lung cancer”；②“Gene: KIF23”；③“Data Type: mRNA和DNA copy number”；④“Analysis Type: Cancer *vs* Normal Analysis”；⑤临界值设定条件（*P* value＜1E-4, fold change＞2, gene rank=top 10%）。选择柱状图展示结果。

### 1.2 *Kaplan*-*Meier* Plotter进行患者生存周期分析

 利用<italic>Kaplan</italic>-<italic>Meier</italic> Plotter数据库（<a href="http://kmplot.com/analysis/" target=_blank>http://kmplot.com/analysis/</a>）的NSCLC数据集进行在线生存分析。筛选条件如下：①“Cancer: Lung Cancer”；②“Gene: KIF23”；③“Survival: OS”。

### 1.3 统计学方法

正常组织与NSCLC病例组之间KIF23表达的差异采用*t*检验。KIF23表达与NSCLC预后的关系采用*Kaplan*-*Meier*模型分析。所有数据采用SPSS 16.0进行统计学分析，以双侧*P*＜0.05为差异有统计学意义。

## 2 结果

### 2.1 KIF23在常见肿瘤类型中的表达结果

Oncomine数据库中共收集了447项不同类型的研究结果（图 1），其中关于KIF23表达有统计学差异的研究结果有67项，KIF23表达增高的研究有64项，表达降低的研究有3项。

**1:**
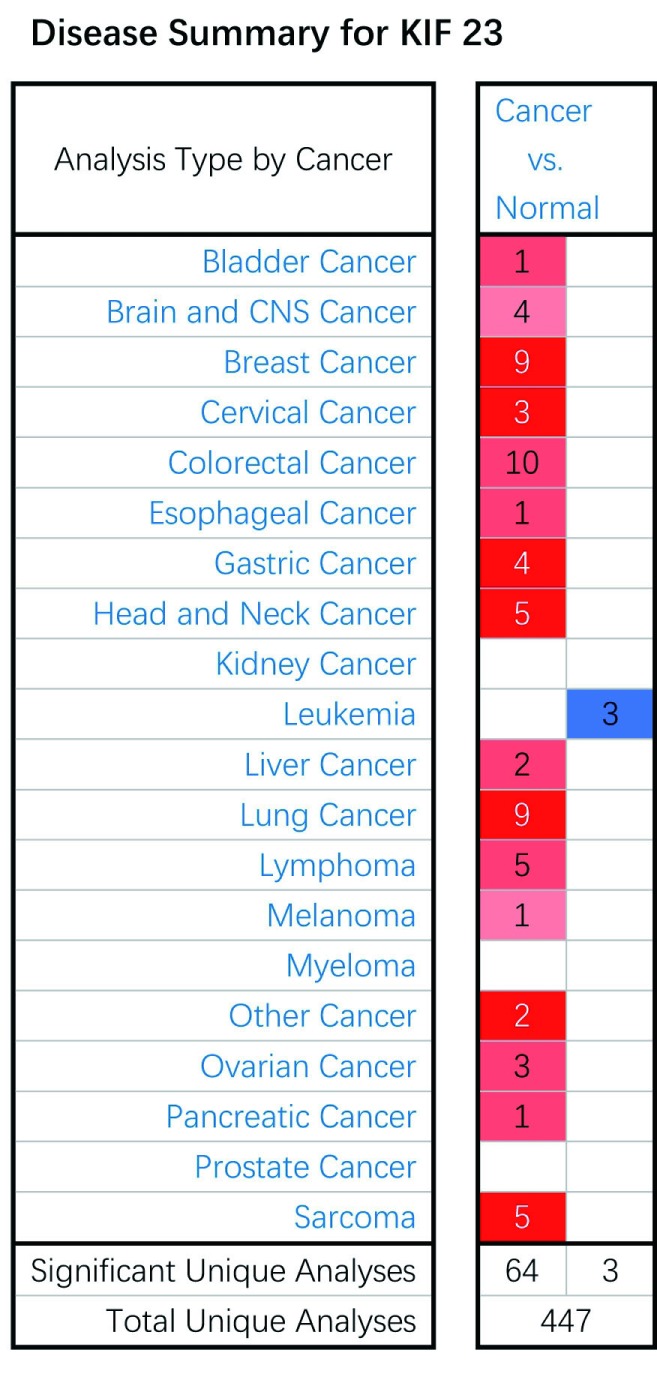
KIF23在Oncomine数据库中所有肿瘤研究中的表达 Expression data for KIF23 in a variety of normal and cancerous human tissues in database Oncomine

### 2.2 KIF23在NSCLC中的表达结果

在Oncomine数据库中我们发现，自2000年开始，共有16项研究涉及KIF23在NSCLC组织和正常组织中的表达（图 2），共有1, 189个样本，包括肺腺癌、鳞癌及大细胞肺癌与正常组织比较。文章分别发表于*Nat Med* ^[10]^、*Genome Res*^[11]^、*Cancer Res*^[12, 13]^、*PLoS One*^[14, 15]^、*BMC Genomics*^[16]^、*Bioinformatics*^[17]^、*Proc Natl Acad Sci U S A*^[18, 19]^和*Am J Pathol*^[20]^。在Oncomine数据库中荟萃分析16项研究结果发现，与对照组相比，*KIF23*基因在所有差异表达基因中其中位数值排名为635.5，*P*=4.14E-8，提示KIF23在NSCLC中高表达。

**2:**
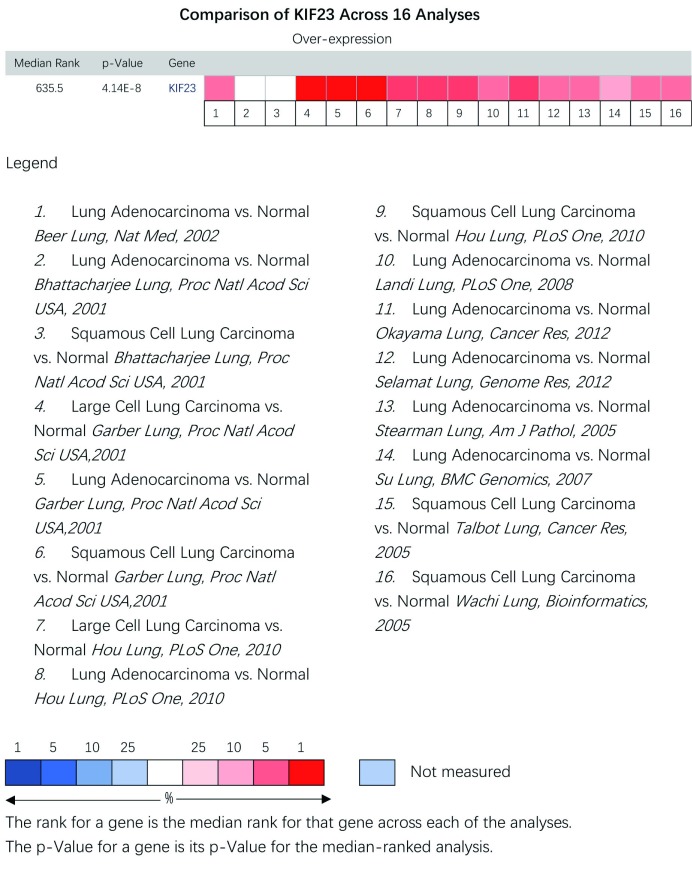
KIF23在Oncomine数据库中非小细胞肺癌中的表达。1-16分别表示16项研究结果，红色越深表示*KIF23*基因在该芯片中表达越高。 Expression of KIF23 in NSCLC in the studies identified in the Oncomine database. 1-16 represent the 16 studies on the expressions of KIF23 in NSCLC. Darker red indicates higher *KIF23* expression in the chips.

### 2.3 KIF23在不同NSCLC研究芯片中的表达差异

图 3所示为Oncomine数据库中KIF23在不同NSCLC研究芯片中的表达结果。分别在Beer等^[10]^、Okayaman等^[12]^、Stearman等^[20]^、Garber等^[18]^和Hou等^[14]^这5项研究中，KIF23在NSCLC中的表达量高于正常组（*P*均＜0.001）。

**3:**
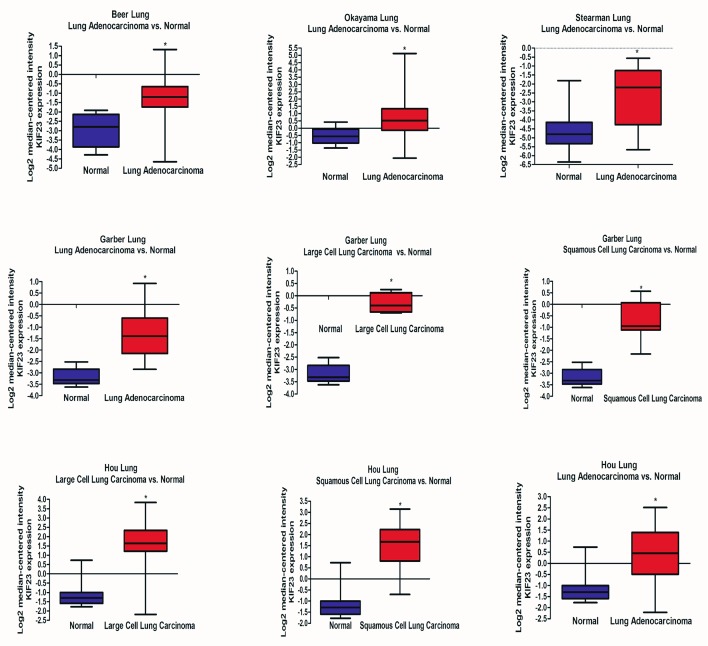
Oncomine数据库中KIF23在不同NSCLC研究芯片中的表达。^*^：*P*＜0.05。 Expression of KIF23 in NSCLC in different studies identified in the Oncomine database. ^*^: *P* < 0.05.

### 2.4 KIF23与NSCLC患者预后的关系

*Kaplan*-*Meier* Plotter数据结果显示：KIF23表达水平对患者的总生存时间有着显著影响。与低表达组相比，KIF23高表达组NSCLC患者的总生存时间显著降低。进一步亚组分析发现，KIF23表达水平对肺腺癌患者预后有显著影响，而在鳞癌患者中，其表达水平对预后无显著影响（图 4）。

**4:**
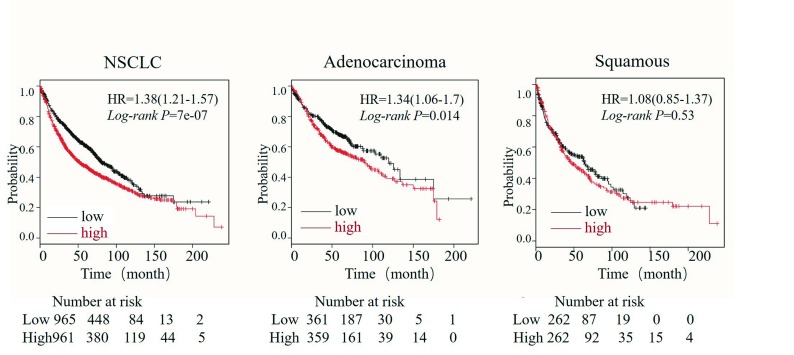
KIF23表达与NSCLC预后之间的关系 Relationship between the expression of KIF23 and the prognosis of NSCLC

## 3 讨论

肺癌是目前全球发病率和死亡率最高的恶性肿瘤之一。并且流行病学统计发现肺癌中的肺腺癌发病率越来越高，已经取代肺鳞癌成为发病率最高的NSCLC^[21]^。长期以来，NSCLC的预后一直较差，直到近十年来，随着分子生物学技术的进展，陆续发现了*EGFR*、*KARS*、*ALK*等一批肺腺癌驱动基因^[22]^，进而开发出一些靶向治疗药物，极大地改善了肺腺癌患者的预后^[23]^，然而这些基因的突变仅存在于肺腺癌当中的部分患者中。因此，寻找NSCLC发生、发展的关键分子或靶点，对于开发治疗肺癌的新的靶向药物具有重要的理论和临床意义，一直是国内外研究的热点。

驱动蛋白（kinesin）是一类能利用ATP水解所释放的能量驱动自身所携带的货物分子沿微管运动的马达蛋白，与细胞内物质运输有关，参与囊泡、细胞器、染色体及RNA结合蛋白等的转运，在胞内物质转运及有丝分裂过程中起着重要的作用。到目前为止，在人和小鼠体内共发现45种驱动蛋白，它们分属14个蛋白家族，根据马达结构域在重链上的位置不同，将驱动蛋白划分为3种类型：马达结构域在C末端的C-kinesin、马达结构域在中间的M-kinesin和马达结构域在N末端的N-kinesin^[24]^，大多数的驱动蛋白是N-kinesin。KIF23属于N-kinesin，定位于细胞胞浆和胞核，与细胞的分化增殖能力有关^[25]^。研究发现，在多种肿瘤如神经胶质瘤、乳腺癌及胃癌中均存在KIF23高表达现象。乳腺癌中KIF23过表达与乳腺癌的肿瘤分级、侵袭性及预后显著相关^[6]^。脑神经胶质瘤细胞KIF23的高表达可能与转录激活有关，体内和体外实验证实，敲除KIF23可以显著抑制胶质瘤细胞的增殖能力^[9]^。此外，在胃癌中也发现，KIF23在mRNA水平显著升高，并且与较差的预后相关^[8]^。Kato等^[26]^研究发现，NSCLC组织中，特别是腺癌组织中KIF23表达明显增高，且高表达患者通常预后比较差。我们通过Oncomine数据库研究，亦同样发现KIF23在多种肿瘤中具有表达增高趋势。

 尽管多数研究发现KIF23在多种肿瘤包括NSCLC中高表达，但由于独立研究存在样本量小易导致抽样误差等原因，缺乏较高的可信度。Oncomine数据库是目前全球最大的基因芯片数据库和整合数据挖掘平台，注册账户后可用于挖掘肿瘤相关基因的信息。我们首先通过Oncomine数据库挖掘<italic>KIF23</italic>基因在结直肠癌、乳腺癌、肺癌等常见肿瘤表达信息，结果发现，在有统计学差异的67项研究中，64项研究表明KIF23在常见肿瘤中处于高表达状态。进一步利用Oncomine数据库荟萃分析了肺癌芯片检测结果，同样证明，在超过1, 000例的标本中，KIF23在NSCLC组织中高表达，尤其是在肺腺癌中高表达。The <italic>Kaplan</italic>-<italic>Meier</italic> Plotter数据库（<a href="http://kmplot.com/analysis/" target=_blank>http://kmplot.com/analysis/</a>）是一个目前世界上最广为接收的预后相关的在线分析数据库，涵盖了2, 437例肺癌样本，可对54, 675个基因进行相关预后分析，并得出真实可信客观的结果。本文首次通过KM plotter数据库发现了KIF23在NSCLC中的预后价值。结果显示，KIF23的表达量与NSCLC总体生存率明确相关，KIF23高表达患者的总生存时间显著降低。进一步亚组分析发现，KIF23表达水平对肺腺癌患者预后有显著影响，而在鳞癌患者中，其表达水平对预后无显著影响。KIF23的高表达可能影响肿瘤的发生，或许由于驱动蛋白家族表达的过度能够影响有丝分裂的正常进行，并导致分裂阻滞或异常分裂的发生，从而产生非整倍体细胞，最终引起肿瘤的发生。我们的所有数据都来自基因芯片，研究方法一致，且包含了迄今为止最大的样本量，去除了因为样本量的问题导致的误差，增加了结论的可信度。

综上所述，我们通过对NSCLC组织中KIF23相关信息的深入挖掘，提出KIF23在NSCLC组织中高表达，且与NSCLC预后相关。采用数据库进行大样本分析，可以避免单一研究样本量过小产生的误差，对临床治疗提供重要理论依据。KIF23在NSCLC疾病发展中的具体作用机制，未来需要进一步的实验来证明。
